# Draft Genome Sequence of *Klebsiella pneumoniae* OK8, a Multidrug-Resistant Mouse and Human Pathogen

**DOI:** 10.1128/genomeA.01018-17

**Published:** 2017-09-14

**Authors:** Easa Nagamalleswari, Valakunja Nagaraja

**Affiliations:** a Department of Microbiology and Cell Biology, Indian Institute of Science, Bangalore, India; b Jawaharlal Nehru Centre for Advanced Scientific Research, Bangalore, India

## Abstract

We report here the draft genome sequence of *Klebsiella pneumoniae* OK8, a multidrug-resistant strain which was isolated in 1976 from a human and is known to be a mouse pathogen.

## GENOME ANNOUNCEMENT

*Klebsiella pneumoniae*, a Gram-negative gammaproteobacterium belonging to *Enterobacteriaceae*, is currently one of the most threatening pathogens due to the emergence of a large number of drug-resistant strains. *K. pneumoniae* exhibits a large diversity, having distinct groups of pathogenic and nonpathogenic serotypes (1). A number of *K. pneumoniae* strains, including drug-resistant clinical isolates, have been sequenced and annotated. Here, we describe the draft genome sequence of *K. pneumoniae* strain OK8, a human pathogen used in the 1980s for antibiotic testing and that is known to cause infection in mice (2). The strain has been used for the isolation of the restriction enzyme KpnI by various groups. We found the organism to be resistant to several antibiotics.

*K. pneumoniae* OK8 was cultured, and total genomic DNA was extracted. Genome sequencing was carried out by Illumina MiSeq (SciGenom). Paired-end libraries were prepared and sequenced. The sequencing reads were assembled into genomic contigs using tools, namely, the A5 pipeline (3), Edena (4), MaSuRCA (5), and SPAdes (6). We found 5 contigs for the bacterial sample, and all contigs were merged using CISA (7). Sequence annotation was carried out with the contigs using the Glimmer-MG program (8). The predicted open reading frames (ORFs) were annotated using our in-house pipeline, CANoPI (Contig Annotator Pipeline). The genome size was found to be 5,768,520 bp, with 57.28% G+C content, having 5,415 coding sequences (CDSs) and 103 tRNAs.

Infections caused by *K. pneumoniae* are rampant throughout the world. Moreover, *K. pneumoniae* is emerging worldwide as a major cause of bacteremia and hospital-borne drug-resistant infections. Strain OK8 has adapted or acquired many mechanisms of antibiotic resistance. Antibiotic susceptibility testing revealed that the strain is resistant to ampicillin, chloramphenicol, kanamycin, and tetracycline but sensitive to aminoglycosides, *viz* gentamicin and streptomycin. The strain harbors a thick polysaccharide capsule thought to be a significant virulence factor, possibly to avoid phagocytosis during infection. The pathogenic and multidrug-resistant features of the strain require further investigations to understand its adaptation and evolution as an opportunistic pathogen.

### Accession number(s).

This whole-genome shotgun project has been deposited in DDBJ/EMBL/GenBank under accession no. MZXR00000000. The version described in this paper is the first version, MZXR01000000.
